# Case report: Migrating temporary epicardial pacing wire eroding into an ascending aortic aneurysm

**DOI:** 10.1016/j.ijscr.2023.108451

**Published:** 2023-06-30

**Authors:** Aaron E. Tipton, Adam Daly, Faisal G. Bakaeen

**Affiliations:** Cleveland Clinic, Department of Thoracic and Cardiovascular Surgery, Cleveland, OH, United States of America

**Keywords:** TEPW migration, Cardiac surgery, Ascending aorta

## Abstract

**Introduction:**

Temporary epicardial pacing wires (TEPW) are commonly placed during cardiac surgery, and a known complication is the migration into visceral and vascular structures. Previous reports have identified TEPW migrating into the ascending aorta. These cases were managed conservatively with the initiation of antithrombotic medications and surveillance. We report the first case of TEPW migration associated with an ascending aortic aneurysm and the operative management.

**Case presentation:**

A 73-year-old man with a history of aortic valve replacement (AVR) and coronary artery bypass grafting (CABG) in 2009 presented to the outpatient clinic for re-operative consideration due to severe bioprosthetic aortic stenosis, ascending aortic aneurysm, and multi-vessel coronary artery disease with occlusion of previous graft. He was incidentally found to have a TEPW eroding into his ascending aorta on pre-operative imaging. He was taken to the operating room for an AVR, ascending aorta replacement, and CABG. The TEPW was removed during the re-operation and the patient recovered well.

**Clinical discussion:**

This is the first reported case of TEPW migration into an aneurysmal ascending aorta and the operative management. The patient tolerated the procedure well and was discharged home. Pre- and intra-operative images were obtained of TEPW extending into the lumen of the ascending aorta. If the patient did not have additional operative indications, conservative management could have been considered with antithrombotic medications and surveillance.

**Conclusion:**

TEPW migration is a rare complication and requires special considerations with balancing risk for intervention.

## Highlights

• TEPW migration into an ascending aortic aneurysm
• CT and Intra-operative imaging of TEPW extending into aortic lumen
• Operative management of TEPW migration into the ascending aorta

## 1 Introduction

**Fig. 1:**
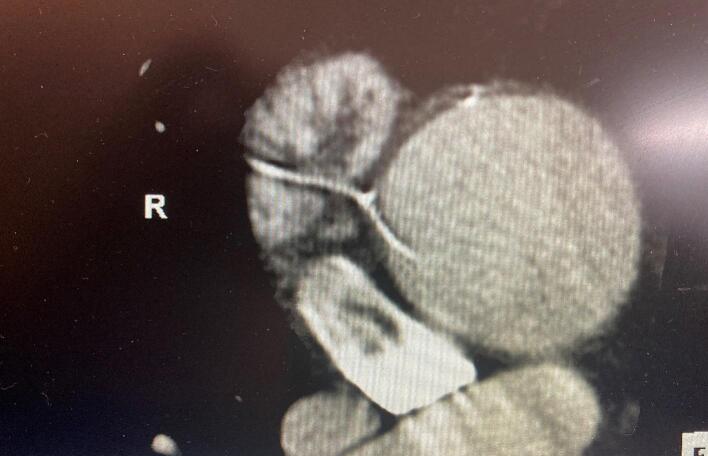
CT Imaging of Intra-Aortic TEPW.

**Fig. 2:**
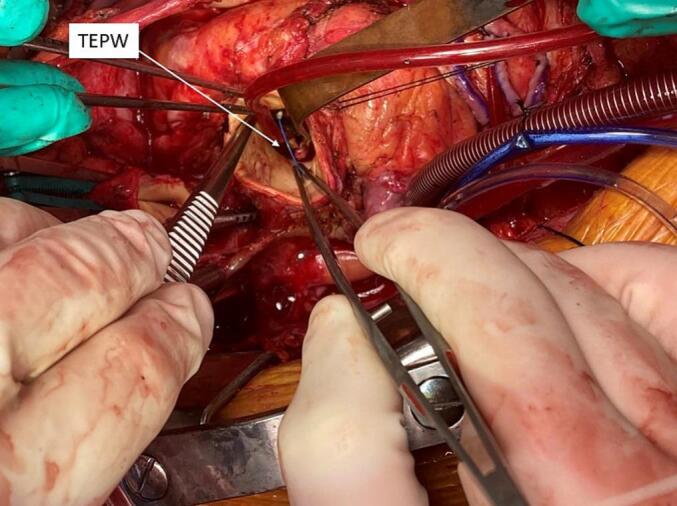
Intra-operative TEPW.

Commonly during cardiac surgery temporary epicardial pacing wires (TEPW) are placed to help treat post-operative arrhythmias. They can be used in a unipolar or bipolar arrangement with atrial leads usually attached to the right atrial appendage and ventricular leads to the right ventricle anterior or diaphragmatic surface. The bare, uninsulated end of the wire is embedded or affixed with a fine suture in direct contact with the myocardium. The wire is tunneled, through the skin, and externalized in the subcostal area where it can be attached to a pacing box. While the patients are hospitalized, they remain and nearing discharge removed via gentle traction or cutting at skin level [1]. The use of TEPW and removal are associated with very low risk [2]. Complications of TEPW are hemopericardium, infection, and migrations with invasion into visceral organs and vascular structures [3]. Here we present a novel case of TEPW migration into an aneurysmal ascending aorta where imaging findings are correlated with intraoperative findings. This case report was reported in line with SCARE criteria [4] (Fig. 1, Fig. 2).

## 2 Presentation of case

We present a 73-year-old man with a past medical history of CABG and aortic prosthetic value placement in 2009, ascending aortic aneurysm, coronary artery disease, SMA stenosis, hypertension, and hyperlipidemia who was evaluated at the outpatient clinic in the winter of 2022 for severe bioprosthetic aortic stenosis, multi-vessel coronary artery disease, and an ascending aortic aneurysm of 4.8 cm. His aortic valve peak gradient was 97 mmHg and the mean gradient was 54. He also had severe and moderate stenosis of his right coronary artery (RCA) and left anterior descending artery, respectively. The previous vein graft to his RCA was occluded. Pre-operative imaging demonstrated a wire structure extending into the ascending aorta via the posterior wall. (Image 1) The wire did not track back to the chest wall on imaging but appeared to rest adjacent to the entry point in the aorta. The patient reported no infectious or atheroembolic history. He was taken to the operative room and underwent a redo bioprosthetic AVR, ascending aorta replacement, and CABG. The proximal ascending aorta was incised with immediate findings of TEPW extending through the posterior aortic wall into the lumen just superior to the noncoronary aortic sinus. (Image 2) It was not involved in the aneurysmal portion of the aorta. The wire was easily removed sharply. No thrombus formation was seen on the wire. The aortic valve and ascending aorta were replaced. Bypass grafts were performed on posterior descending, posterior left ventricular, and left anterior descending arteries with greater saphenous veins and in-situ left internal mammary artery, respectively. The patient tolerated the procedure well. His postoperative course was unremarkable with discharge home on postoperative day 7.

## 3 Discussion

Retained TEPW migration can result in clinical complications, but most patients remain asymptomatic. We presented a case of an asymptomatic, incidental finding of a retained TEPW penetrating through the proximal ascending aortic wall into the aortic lumen. The patient was then taken to the operating for ascending aortic replacement, AVR, and CABG. The TEPW was removed at this time. It is unclear what caused the migration of TEPW but multiple accounts in the literature have described the incidental migration into cardiac and vascular structures [5]. Previously described cases of TEPW transaortic migration were managed conservatively with antithrombic/anticoagulation medications and close follow-up [6]. If our patient did not have other indications for operative intervention, his TEPW may have been observed with surveillance imaging and initiation of antithrombotic medications. He would have a lifelong increased risk of stroke with the wire material in his ascending aorta. Currently, there is no long-term follow-up data on the conservative management of TEPW migration into the aorta. Ultimately, our patient tolerated a re-operative well and TEPW was removed.

## 4 Conclusion

From our limited experience, cases of TEPW migration need to be addressed on an individual basis and it would be difficult to recommend removal in all cases with cardiac re-operation having significant risks of morbidity and mortality. Additionally, some wires may be removed with endovascular approaches [5]. In the case presented, the patient had additional indications for a re-operation. Furthermore, it may also be beneficial for observation of such findings with surveillance imaging and initiation of antiplatelets and anticoagulation to reduce thrombosis formation [6]. TEPW migration is a rare complication, which requires special considerations and balancing risk for intervention.

## Consent

Written informed consent was obtained from the patient for the publication of this case report and accompanying images. A copy of the written consent is available for review by the Editor-in-Chief of this journal on the request.

## Ethical approval

Case reports are exempt from institutional review board/ethical approval by Cleveland Clinic. This case report was exempt from institutional review board approval. All identifying personal health information has been removed from the presentation.

## Sources of funding

None.

## Author contribution

Aaron E. Tipton, MD – Writer.

Adam Daly, MD – Editor.

Faisal G Bakaeen, MD – Editor, Supervisor.

## Research registration number

No Registry.

## Guarantor

Dr. Faisal G Bakaeen, MD.

## Conflict of interest statement

The authors have no financial or personal disclosures related to this work.

## References

[1] Richards C. CTSNet Step-by-Step Series: Pacing Wires. doi:10.25373/ctsnet.7128545.

[2] Mishra P.K., Lengyel E., Lakshmanan S., Luckraz H. (2010). Temporary epicardial pacing wire removal: is it an innocuous procedure?. Interact. Cardiovasc. Thorac. Surg..

[3] Wald G., Van Y.V.R., Pain K.J., Otterburn D.M. (2020). Retained temporary epicardial pacing wires: a systematic review and treatment algorithm. Ann. Plast. Surg..

[4] Agha R.A., Franchi T., Sohrab C., Mathew G., Kirwan A., Thomas A. (2020). The SCARE 2020 guideline: updating consensus surgical case report (SCARE) guidelines. Int. J. Surg..

[5] Hua J.D., Ali S.S., Reddy V., Patel S.J. (2020). Wandering atrial pacemaker wire: migration of a temporary epicardial pacing wire into the left heart. JACC Case Rep..

[6] Kahaly O.R., Patel D., Augostini R.S., Rushing G.D., Houmsse M.M. (2020). Intra-aortic migration of a clipped epicardial pacing wire. Tex. Heart Inst. J..

